# Apicomplexan Protozoa Responsible for Reproductive Disorders: Occurrence of DNA in Blood and Milk of Donkeys (*Equus asinus*) and Minireview of the Related Literature

**DOI:** 10.3390/pathogens10020111

**Published:** 2021-01-22

**Authors:** Stefania Perrucci, Lisa Guardone, Iolanda Altomonte, Federica Salari, Simona Nardoni, Mina Martini, Francesca Mancianti

**Affiliations:** Department of Veterinary Sciences, University of Pisa, Viale delle Piagge 2, 56124 Pisa, Italy; lisa.guardone@for.unipi.it (L.G.); altomonte@vet.unipi.it (I.A.); federica.salari@unipi.it (F.S.); simona.nardoni@unipi.it (S.N.); mina.martini@unipi.it (M.M.)

**Keywords:** donkeys, *Neospora caninum*, *Toxoplasma gondii*, *Theileria equi*, milk, blood, DNA

## Abstract

Donkeys may be susceptible to many pathological agents and may act as carriers of pathogens for other animal species and humans. This study evaluated the occurrence of potentially abortifacient apicomplexan protozoa DNA in blood and milk samples collected at different time periods during lactation (1, 6, and 10 months) from 33 healthy dairy jennies. A total of 73 blood and 73 milk samples were used for DNA extraction and analysis. Blood specimens from 11/33 (33%) jennies scored positive for *Theileria equi*, while milk samples scored negative. Blood and milk of 3/33 jennies yielded DNA of *Toxoplasma gondii* at 6 months (n. 1) and 10 months (n. 2) after parturition. *Neospora caninum* DNA was found in four milk and in five blood samples only at one month after parturition. This study is the first report about the presence of *N. caninum* DNA in milk of naturally infected jennies. Moreover, the excretion of *N. caninum* DNA in some of these jennies at 30 days from the parturition may suggest a possible occurrence of an endogenous cycle, while the presence of *T. gondii* DNA in the milk collected at 6 and 10 months after parturition may be suggestive of a discontinuous excretion.

## 1. Introduction

Donkeys (*Equus asinus*) differ from horses, being more stoical in their behavior and showing limited fear response and a great adaptability to different environmental conditions. They have narrow, vertical hooves that are suitable for rocky areas, and they can tolerate hot dry conditions, often in areas with low-quality forages. For all these reasons, donkeys are considered a more rustic species than horses [1]. Donkeys are worldwide bred for both productive (work and milk) and recreational aims. In recent years, donkey breeding has increased, also following the preservation and protection of autochthonous breeds [1]. However, the studies about the occurrence of pathogens of the donkey are still scant, being that this animal species is considered to not require much care. For these reasons, donkey health problems are neglected [2]. Nevertheless, donkeys may be susceptible to many pathological agents and, being their breeding is often promiscuous [3], they could also act as carriers of pathogens for other animal species and humans, when zoonotic agents are involved. In Tuscany (central Italy), Amiatina is an endangered autochthonous donkey breed, reared for both milk production and onotherapy; that is a donkey-assisted therapy aiming at improving body functions, activities, and participation in subjects affected by various disabilities [4].

Donkey milk is produced for human consumption, mostly intended for allergic infants and/or elderly people, and for cosmetic use. Therefore, microbiological controls to certify milk quality are required by the European law (Regulation EC 853/2004) [5] and routinely achieved in dairy donkey farms. However, to the best of our knowledge, data concerning the occurrence of potentially abortifacient apicomplexan protozoa in this animal species are scant, and the occurrence of the DNA of these protozoa in donkey milk has been scarcely investigated.

Indeed, among these agents, available data are only referred to *Toxoplasma gondii* [6], a zoonotic protozoan with cats as definitive hosts. The infection can be acquired by the ingestion of infected raw meat and milk, as well as from sporulated oocysts, and it has been reported worldwide in donkeys [3,6,7,8,9,10,11]. Although *T. gondii* is one of the main abortifacient agents, the infection in horses is usually subclinical. Nevertheless, atypical clinical signs such as fever, ataxia, retinal degeneration, encephalomyelitis, and abortion or stillbirth in pregnant equids have been described [7].

*Neospora caninum* is an Apicomplexan protozoan parasite closely related to *T*. *gondii*. Dogs act as final hosts and different mammals, including equids, are intermediate hosts, with cysts in the central nervous system and in the muscular tissue. This protozoan is a well-known abortifacient agent, with a large impact on cattle breeding, but it has been recently recorded as a cause of abortion and neonatal diseases also in horses [12,13,14]. *N*. *caninum* infection has been reported in donkeys worldwide, with different seroprevalences [10,15,16,17]. Although direct evidence did not suggest that abortion in donkeys is caused by *N*. *caninum*, jennies with an history of miscarriage showed significantly higher seroprevalence values for *N. caninum* than controls [16,18].

*Theileria equi* is the main agent of equine piroplasmosis in Tuscany [19]. This protozoan species is responsible for a tick-borne disease, causing important economic losses in equine breeding. Acute infection is reported to lead to abortion in mares [20,21,22,23]. However, recent findings suggested that transplacental transmission of *T. equi* is not common, and that this parasite should not be considered as a substantial cause of equine abortion [24]. Conversely, piroplasmosis develops as a chronic disease in infected donkeys [25], and this animal species may act as a reservoir for *T. equi* infections in horses. The infection has been described worldwide [26,27,28], and it has also been reported in donkeys from Italy [29,30,31,32], frequently associated with a specific clinical presentation and production losses [33].

The aims of this study were (i) to evaluate the occurrence of DNA of these selected protozoa in blood and milk samples collected at different time periods during lactation, from multiparous healthy dairy jennies of an autochthonous (Amiatina) breed from Tuscany, central Italy; and (ii) to provide a short review of the state of the art on the occurrence of these protozoa in milk.

## 2. Results

Blood and/or milk samples from 19 jennies scored positive for at least one of the selected protozoans (Table 1).

Blood specimens from 11/33 (33%) jennies scored positive for *T. equi*, while milk did not show the presence of this piroplasm DNA. Positive blood samples tested positive at every checking.

Blood and milk of three jennies presented DNA of *T. gondii*, two among them at ten months and the other at six months after parturition.

*N. caninum* DNA was detected in blood samples from five jennies at one month after parturition, whilst the following sampling scored negative. In four of these jennies, *N. caninum* DNA was also detected in the milk at the same lactation time (one month).

All the animals with positive milk specimens showed the presence of parasite DNA in blood.

A coinfection by *T. equi* and *N. caninum* was observed in a single jenny only (jenny N. 4, Table 1). More detailed results are reported in Table 1.

## 3. Discussion

The presence of *T. gondii* in milk has been reported since 1948 [34]. In fact, drinking raw milk is considered as a potential risk factor for *T. gondii* infection in human patients [35] and this topic has been reviewed by Boughattas [36]. Bovine milk had previously been considered a negligible source of infection [37], while milk from buffalo tested positive for DNA [38] and for both DNA and intact parasites [39]. However, cattle and buffaloes are considered as resistant species to *T. gondii* infection [40], and the results of the study of Dehkordi et al. [39] have been questioned [41].

Human clinical toxoplasmosis has been mostly attributed to raw goat milk consumption [42,43,44,45]. Caprine milk resulted positive for parasite DNA [38,46,47,48,49,50,51,52,53], and for both DNA and viable tachyzoites also [39,41], while in another study goat milk tested negative in contrast to sheep and camels [54]. Milk of *T. gondii* infected camels was successfully bioassayed in mice [55], in cats and cells [39], and in mice and kittens [56]. The presence of *T. gondii* DNA in camel milk has been reported in several studies [38,39,54], even though in another study all specimens tested negative [53]. The literature dealing with potential infection of ovine milk has been recently reviewed [57], and *T. gondii* DNA and viable tachyzoites occurrence has also been reported [38,39,48,53,54,58,59,60,61,62,63,64]. Camossi et al. [59] reported the presence of *T. gondii* DNA in milk from naturally infected ewes and detected *T. gondii* DNA twice in the milk of two sheep, suggesting a possible resurgence of tachyzoites from cysts, which can circulate again and be excreted through the milk. These findings were recently corroborated by a longitudinal investigation showing a discontinuous parasite excretion via goat milk [65]. Thus, despite the low number of jennies here examined, the presence of *T. gondii* at six and nine months after parturition in some jennies in this study would seem in agreement with these latter observations.

Data about the occurrence in donkey milk of DNA of the other abortifacient *Apicomplexan protozoa* considered in this study are lacking. To the best of our knowledge, this is the first report on the occurrence of *N. caninum* DNA in donkey milk. In chronically infected hosts, *N. caninum* may reactivate during pregnancy, and the consequent parasitemia may allow transplacental infection [66]. The endogenous cycle, reported as probably not able to maintain the parasite in dogs, is considered as the preeminent route of infection in cows [67], and it has been recently observed also in ewes [68]. Furthermore, a vertical post-natal transmission via milk was reported in a murine model [69,70], in 1 out of 51 puppies [69] and in cattle [71]. The presence of *N. caninum* DNA was demonstrated in cow milk, also [72]. The possible oral infection via colostrum was firstly demonstrated in experimentally infected calves within a few hours from the birth [73], and then confirmed in calves aged up to one week administered with colostrum added with tachyzoites, but not with milk or colostrum taken from naturally infected dams. For this reason, colostrum and milk were not considered as an important natural route for the transmission of *N. caninum* in cattle so far [74]. Anyway, the finding of *N. caninum* DNA in blood and milk of 4 out of the 22 samples drawn one month after parturition would suggest at least a possible occurrence of an endogenous cycle also in donkeys, with a recurrent parasitemia during pregnancy.

The prevalence rate of blood piroplasm DNA (33%) matches perfectly with a previous investigation from the same breeding [32], and the negative results for piroplasm DNA in donkey milk confirms the lack of reports dealing with the excretion of *T. equi* DNA via milk. Data about the trans-mammary transmission of piroplasms are scant. This route has been ruled out in puppies born from a bitch chronically infected by *Babesia gibsoni* [75], while this possibility was not excluded in a dog litter found infected by *Babesia canis* [76]. Colostrum samples from a cow herd endemic for *Theileria orientalis* scored positive by qPCR. However, all calves administered with colostrum tested PCR negative and only one among them appeared antibody positive, suggesting colostrum transfer as a possible mode of infection [77].

## 4. Materials and Methods

### 4.1. Animals and Sampling

A total of 146 blood and raw milk samples (73 and 73, respectively), drawn at the same moment from 33 lactating jennies were used for DNA extraction. The subjects, aged from 4 to 18 years, were semi-extensively reared in a dairy donkey farm near Scarlino (42_ 530 52.59 N 10_ 470 05.52 E, WGS84), Tuscany, Central Italy. The farm produces pasteurized milk for human consumption according to European Union (EU) Regulation 853/2004 [5], and total bacterial count for Enterobacteriaceae, *Escherichia coli*, *Salmonella* spp., *Listeria monocytogens* and *Staphylococcus* spp. is routinely achieved to certify milk safety. All the selected jennies had been living on the farm for many years. Repellent as well as insecticide and other antiparasitic treatment had not been administered to any animal in the 12 months prior to the beginning of this study. No dogs neither cats were present on the farm.

Blood and milk samples were previously collected to evaluate hematological and milk cytological parameters during the whole span of lactation and to characterize genetic polymorphisms of selected animals [78]. For these reasons, samples drawn at about 1, 6 and 10 months after parturition were available. Blood samples (50 mL) were drawn from the jugular vein in sterile tubes with ethylenediaminetetraacetic acid (EDTA). Collected milk and blood samples were maintained a 4 °C until DNA extraction.

As shown in Table 2, 11 animals had a unique blood and milk sampling, 4 jennies had two blood and milk samplings, and from the other 18 animals, blood and milk samples were obtained three times. In total, 22, 20 and 31 blood and milk samples were available at 1 month, 6 months, and 10 months of lactation, respectively (Table 2).

### 4.2. Molecular Analyses

Fifty milliliters of milk were used for DNA extraction as previously reported [6]. Briefly, the sample was concentrated by centrifuge and, to avoid interference by casein, 1 mL of pellet was processed following Psifidi et al. method [79]. Somatic cells were diluted in 200 µL of PBS and submitted to DNA extraction. DNA was extracted from both blood samples and milk somatic cells using the commercial kit DNeasy Blood & Tissue Kit (Qiagen, Milano, Italy), following the procedures reported by the producer. DNA was stored at 4 °C until used as template for PCR assays.

All DNA samples were analyzed through different PCR assays to detect *T. equi*, *N. caninum,* and *T. gondii*. More specifically, piroplasm DNA was searched for as reported by Beck et al. [80]. DNA of *N. caninum* was detected by a PCR carried out as described by Müller et al. [81] and an nPCR for *T. gondii* was performed as described by Jones et al. [82] with slight modifications, as previously described [6].

PCR were performed with Wonder Taq (Euroclone, Italy) in an automated thermal cycler (Gene-Amp PCR System 2700, Perkin Elmer, Norwalk, CT, USA). Sterile distilled water was used instead of DNA in the negative control. PCR products were analyzed by electrophoresis on 2% agarose gel stained with GelRed^®^ Nucleic Acid Gel Stain (Biotium). SharpMass™ 100 Plus Ladder (Euroclone, Milano, Italy) was used as DNA marker.

PCR products obtained from samples found positive for piroplasms were sequenced and analyzed. All sequencing procedures were performed by a commercial laboratory (BMR-Genomics, Padova, Italy). Sequences were assembled and corrected by visual analysis of the electropherogram using Bioedit v.7.0.2 30, then compared with those available in GenBank using the BLAST program (http://www.ncbi.nlm.nih.gov/BLAST) to assign the species.

## 5. Conclusions

This study is the first report about the detection of *N. caninum* DNA in the milk of naturally infected jennies. Although the sample size is very small, the excretion of *N. caninum* DNA at 30 days from the parturition may suggest the possible occurrence of an endogenous cycle, as previously observed in cattle [67]. Similarly, the presence in some jennies of *T. gondii* DNA in the milk collected at 6 and 10 months after their parturition may be suggestive of a discontinuous excretion, as reported for small ruminants [59,65]. Conversely, data from this study may suggest that in lactating jennies *T. equi* DNA is not excreted via the milk, but further studies are needed to confirm this finding.

## Figures and Tables

**Table 1:** Lactating Amiatina jennies reared for milk production in Tuscany (central Italy) which scored positive (19/33 of the examined jennies) at PCR analysis of milk and blood for DNA of Apicomplexa protozoa responsible for reproductive disorders at the different samplings (first, sixth, and tenth month of lactation). All positive jennies were negative for the presence of *Theileria equi* DNA in milk.

N. Jenny	*Toxoplasma gondii* Milk	*Toxoplasma gondii* Blood	*Neospora caninum* Milk	*Neospora caninum* Blood	*Theileria equi* Blood
1	-	-	-	-	+ °
2	-	-	-	-	+ °
3	-	-	-	-	+ °
4	-	-	+ ^§^	+ ^§^	+ °
5	-	-	-	-	+ °
6	-	-	-	+ ^§^	-
7	-	-	+ ^§^	+ ^§^	-
8	-	-	-	-	+ °
9	+ ^	+ ^	-	-	-
10	-	-	-	-	+ °
11	-	-	+ ^§^	+ ^§^	-
12	-	-	-	-	+ °
13	-	-	-	-	+ °
14	-	-	-	-	+ °
15	+ ^	+ ^	-	-	-
16	-	-	-	-	-
17	-	-	+ ^§^	+ ^§^	-
18	-	-	-	-	+ °
19	+ *	+ *	-	-	-

^§^ first sampling (first month of lactation); * second sampling (sixth month of lactation); ^ third sampling (tenth month of lactation); ° all samplings (first, sixth, and tenth month of lactation).

**Table 2:** Number of examined milk and blood samples taken from 33 lactating jennies reared for milk production in Tuscany (central Italy) at the 1st, 6th, and 10th months of lactation, and number of jennies having 1, 2, or 3 samplings.

Milk and Blood Samples			N. Samplings/N. Jennies		
1st month	6th month	10th month	1	2	3
n. 22	n. 20	n. 31	n. 11	n. 4	N. 18

## Data Availability

All data are contained within the article.
